# Effect of Natural and ARV-Induced Viral Suppression and Viral Breakthrough on Anti-HIV Antibody Proportion and Avidity in Patients with HIV-1 Subtype B Infection

**DOI:** 10.1371/journal.pone.0055525

**Published:** 2013-02-20

**Authors:** Sarah K. Wendel, Caroline E. Mullis, Susan H. Eshleman, Joel N. Blankson, Richard D. Moore, Jeanne C. Keruly, Ron Brookmeyer, Thomas C. Quinn, Oliver Laeyendecker

**Affiliations:** 1 Division of Intramural Research, National Institute of Allergy and Infectious Diseases, National Institutes of Health, Baltimore, Maryland, United States of America; 2 Department of Medicine, Johns Hopkins University School of Medicine, Baltimore, Maryland, United States of America; 3 Department of Pathology, Johns Hopkins University School of Medicine, Baltimore, Maryland, United States of America; 4 Department of Biostatistics, School of Public Health, University of California Los Angeles, Los Angeles, California, United States of America; Rush University, United States of America

## Abstract

**Background:**

Viral suppression and viral breakthrough impact the humoral immune response to HIV infection. We evaluated the impact of viral suppression and viral breakthrough on results obtained with two cross-sectional HIV incidence assays.

**Methods:**

All samples were collected from adults in the US who were HIV infected for >2 years. Samples were tested with the BED capture enzyme immunoassay (BED-CEIA) which measures the proportion of IgG that is HIV-specific, and with an antibody avidity assay based on the Genetic Systems 1/2+ O ELISA. We tested 281 samples: (1) 30 samples from 18 patients with natural control of HIV-1 infection known as elite controllers or suppressors (2) 72 samples from 18 adults on antiretroviral therapy (ART), with 1 sample before and 2–6 samples after ART initiation, and (3) 179 samples from 20 virally-suppressed adults who had evidence of viral breakthrough receiving ART (>400 copies/ml HIV RNA) and with subsequent viral suppression.

**Results:**

For elite suppressors, 10/18 had BED-CEIA values <0.8 normalized optical density units (OD-n) and these values did not change significantly over time. For patients receiving ART, 14/18 had BED-CEIA values that decreased over time, with a median decrease of 0.42 OD-n (range 0.10 to 0.63)/time point receiving ART. Three patterns of BED-CEIA values were observed during viral breakthrough: (1) values that increased then returned to pre-breakthrough values when viral suppression was re-established, (2) values that increased after viral breakthrough, and (3) values that did not change with viral breakthrough.

**Conclusions:**

Viral suppression and viral breakthrough were associated with changes in BED-CEIA values, reflecting changes in the proportion of HIV-specific IgG. These changes can result in misclassification of patients with long-term HIV infection as recently infected using the BED-CEIA, thereby influencing a falsely high value for cross-sectional incidence estimates.

## Introduction

HIV incidence estimates are used to monitor the current state of the epidemic and determine the impact of prevention efforts and treatment policy. Longitudinal cohorts can be used to determine HIV incidence [1]; yet, longitudinal cohort studies are expensive and suffer from selection and follow-up biases [2], [3]. Cross-sectional surveys have been used to identify recently-infected patients and estimate population incidence [3]. However, most laboratory methods used for cross-sectional HIV incidence determination misclassify some patients with long-term infection as recently infected, causing inaccurate incidence estimates [4]. One key factor associated with misclassification by cross-sectional incidence assays is viral suppression, both natural and antiretroviral (ARV) drug induced [5]. Self-report of ARV treatment (ART) has been used to exclude patients from being considered recently infected; however, self-report of ART is inaccurate [6]. Some patients who are receiving ART report that they are not on treatment.[7] Other patients receiving ART are not virally suppressed, due to viral resistance or lack of adherence [6]. Furthermore, detection of ARVs in blood can be difficult because of the short half-life of some ARV drugs [6], [8]. In addition, viral breakthrough is another important component that could be affecting cross-section incidence estimates. Viral breakthrough is defined as the reemergence of virus, while receiving ART, to >1000 copies/mL [9]. A summary on these and other issues associated with cross sectional incidence testing can be found in recent reviews.[10], [11], [12] We evaluated the impact of viral suppression and viral breakthrough on results obtained from cross-sectional incidence testing assays.

In this report, we evaluated the impact of viral suppression on the anti-HIV antibody response, measured using two different cross-sectional incidence assays: (1) the proportion of IgG that is HIV specific (measured using the BED capture immunoassay [13] (BED-CEIA), and (2) antibody avidity (measured using a modified enzyme immunoassay). The avidity immunoassay measures the strength of the HIV antibody response [14]. It has been recorded that the avidity response directly correlates with the amount of time an patient has been infected [14]. We evaluated these effects in patients with both natural and ARV-induced viral suppression. We also evaluated the effect of duration of viral suppression and the impact of viral breakthrough on these immune responses.

## Methods

Samples were collected from adults with likely HIV-1 subtype B infection, from the Johns Hopkins HIV Clinical Practice Cohort [15] in Baltimore Maryland, who were infected for at least two years. A study of HIV infected subjects determined that >98% of patients from inner city Baltimore were infected with HIV-1 subtype B virus [16]. All subjects from the elite suppressor cohort at Johns Hopkins University are infected with HIV-1 subtype B virus [17], [18], [19]. This included: (1) 18 patients who were identified as elite suppressors [17] (30 samples); (2) 18 patients receiving ART with one sample before and 2–6 samples (one/year) after ART initiation (72 samples); and (3) 20 virally-suppressed patients who had evidence of viral breakthrough while receiving ART (>400 copies/ml) with subsequent viral suppression (179 samples). All participants provided written informed consent and the study was approved by the Institutional Review Board of the Johns Hopkins University.

The BED-CEIA [20] was performed according to the manufacturer's instructions (Sedia Biosciences Corporation, Portland, OR); with the exception that all samples were run in duplicate. The average normalized optical density (OD-n) was used for the analysis. Since all of patients had documented HIV infection for a minimum of two years, any sample with an OD-n ≤0.8 was considered misclassified. The avidity assay was based on the Genetic Systems HIV-1/HIV-2 EIA +O (enzyme linked immunoassay, BioRad Laboratories, Redmond, WA)[21]. In short, samples were diluted 1:10 in duplicate, and were incubated at 4°C for 30 minutes (initial antibody-binding step). Samples were then incubated for 30 minutes at 37°C with or without the chaotropic agent, diethylamine (antibody disassociation step). For each sample, the avidity index was calculated as: [optical density of the diethylamine-treated well]/[optical density of the non-treated well] ×100. For both the BED-CEIA and avidity assays, all samples from a given patient were run on the same plate to minimize the effect of plate-to-plate variation.

## Results

**Figure 1:**
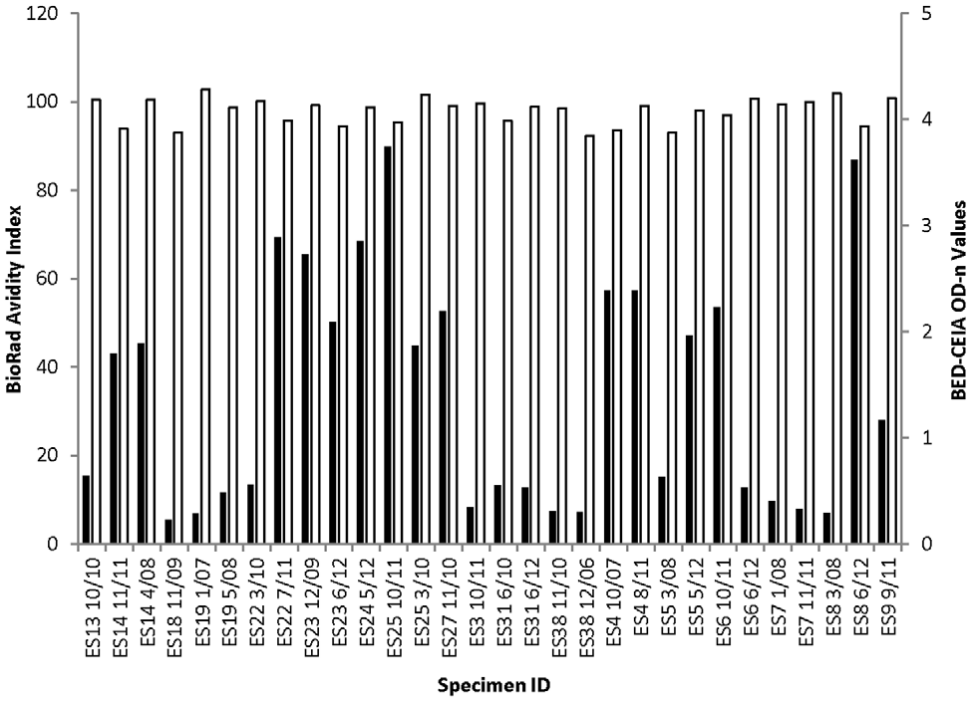
Elite suppressor BioRad avidity index and BED-CEIA OD-n values. Results from 30 samples from 18 elite suppressors (patients who had viral load <50 copies/mL without antiretroviral therapy). The month and year of sample are listed. Black bars denote BED-CEIA OD-n values and white bars denote BioRad avidity index.

**Figure 2:**
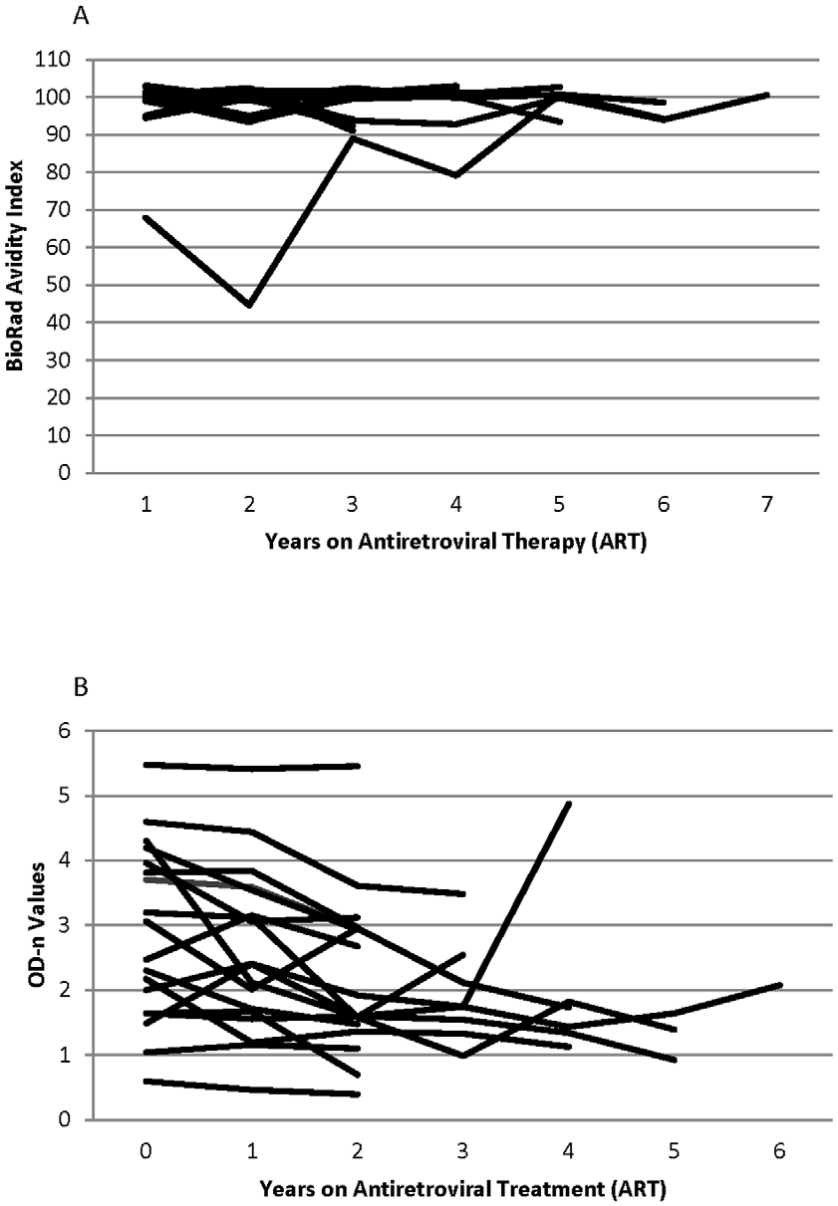
The effect of time after ART-induced viral suppression on antibody avidity and BED-CEIA results. (A) BioRad avidity index values of 18 patients (72 samples) receiving ART with one sample before and 2–6 samples after ART initiation. (B) BED-CEIA normalized optical density (OD-n) values of 18 patients (72 samples) receiving ART, with one sample before and 2–6 samples after documented viral suppression.

For the elite suppressors, the avidity index ranged from 92% to 101% and was well above the threshold of 40% for recent infection [21] (Figure 1). The avidity index was also not affected by longer duration of ART or viral breakthrough (Figure 2B). Of the all 38 patients receiving ART, 26 consistently had avidity index values above 80% for all time points tested. One subject had an avidity index of 70% prior to ART initiation that decreased during the first year of treatment and then increased to and remained above 80% at two years receiving ART (Figure 2B). The second subject had an avidity index of 35% at the first time point (consistent with recent HIV infection) that slowly climbed to above 80% by the last time that the last sample was collected (after 9 years receiving ART). Viral suppression had no detectable impact on antibody avidity.

**Figure 3:**
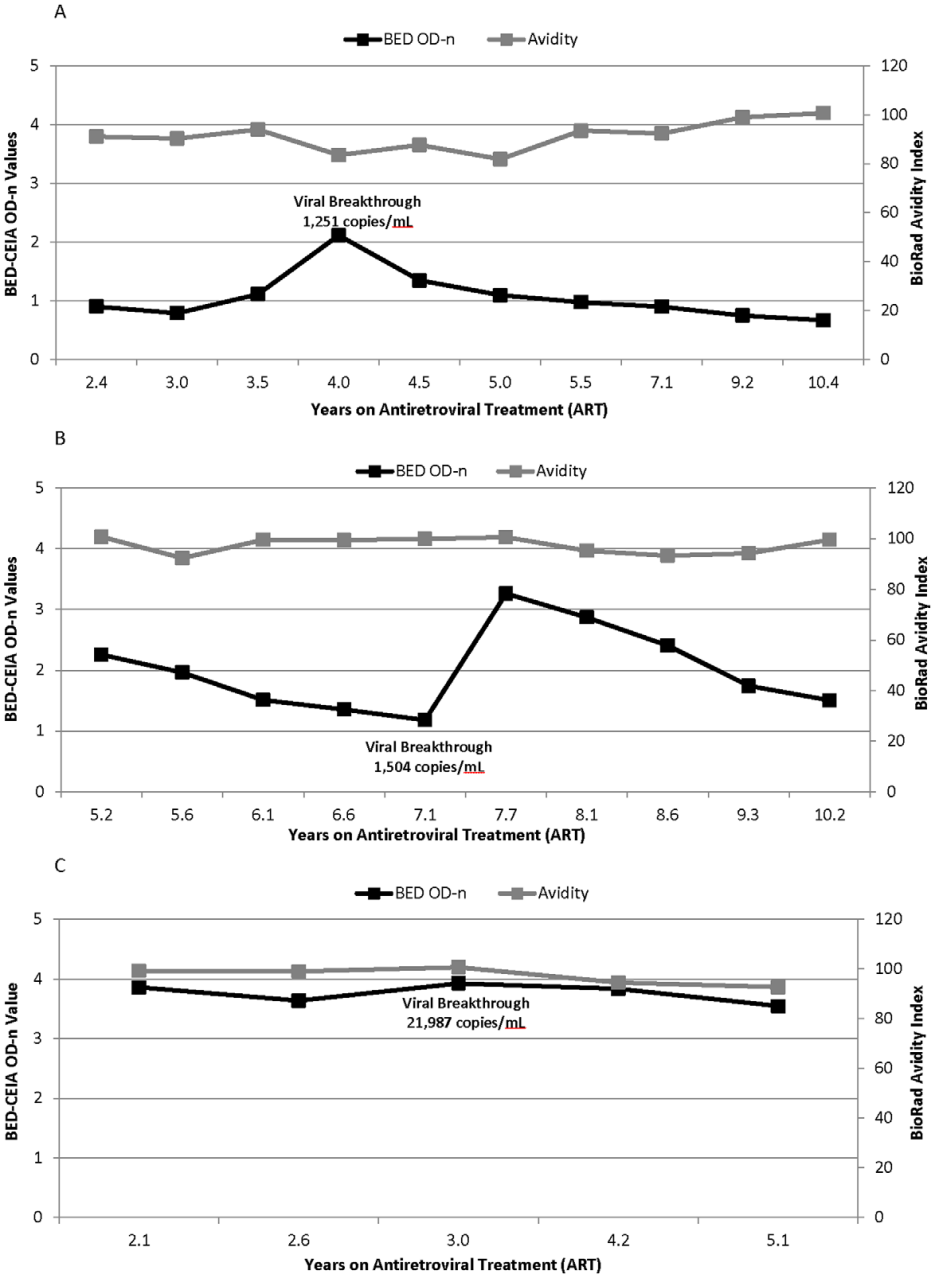
The effect viral breakthrough on BED-CEIA and avidity results. 20 virally-suppressed patients (179 samples) who had evidence of viral breakthrough while receiving ART (>400 copies/ml) with subsequent viral suppression. (A) Individual example of BED-CEIA OD-n values increase with viral breakthrough. Apart for time point listed, all other time points had viral loads <400 copies/ml. (B) Example of the increase in BED-CEIA OD-n values after viral breakthrough time point; results from one patient. Apart for time point listed, all other time points had viral loads <400 copies/ml. (C) Example of BED-CEIA OD-n values that showed no change with viral breakthrough. Apart for time point listed, all other time points have viral loads <400 copies/ml.

In contrast, viral suppression had a significant impact on results obtained with the BED-CEIA; this was observed for patients with both natural and ARV-induced viral suppression. Ten of 18 elite suppressors had BED-CEIA values ≤0.8 OD-n (Figure 1). After ART initiation, BED-CEIA values decreased in 14 of 18 patients, with a median decrease of 0.42 OD-n per year of treatment (range 0.10 to 0.63) (Figure 2A). In those with viral breakthrough, three patterns of BED-CEIA values were observed. In 9 of 20 patients, values increased at the time of viral breakthrough by a median of 0.52 OD-n, and returned to pre-breakthrough values when viral suppression was re-established (Figure 3A). In 8 of 20 patients, values increased at the visit after viral breakthrough (mean 322 days after breakthrough, range 0.8–2.8), with a median increase of 0.81 OD-n (Figure 3B). In the remaining three subjects, there was no change in BED-CEIA values with viral breakthrough (Figure 3C).

## Discussion

In our study, we found that viral suppression impacts results obtained with the BED-CEIA, but does not impact results obtained with an antibody avidity assay. BED-CEIA values varied in patients experiencing viral suppression and viral breakthrough. Elite suppressors had naturally low BED-CEIA values; 10 of 18 elite suppressors were misclassified as recently-infected using this assay. ART use was also associated with decreasing OD-n values. The observed effect compromises use of the BED-CEIA for accurately assessing HIV incidence. As the number of HIV-infected patients accessing ART increases world-wide, and patients receiving ART survive longer, the frequency of long-term infected patients who are misclassified as recently infected by the BED-CEIA would be expected to increase over time.

The finding that ART-induced viral suppression is associated with BED-CEIA misclassification has been reported previously [4], [20], [22]. Our study defines a rate of decrease in BED-CEIA OD-n values after ART initiation, and demonstrates that BED-CEIA results do not change in some patients even after prolonged ART. We observe that the BED-CEIA values increase in the majority of patients with viral breakthrough, and that there is a temporal relationship between the increase of the BED-CEIA value and the increase in viral load. These findings support the use of viral load testing as a marker of ART use in combination with additional assay, including the BED-CEIA for analysis of cross-sectional incidence [5], [23], [24], [25].

Similar to results observed with the Vironostika-Less Sensitive enzyme immunoassay (Vir-LS), the BED-CEIA misclassifies a majority of samples from elite suppressors [22]. This finding supports previous results suggesting that the BED-CEIA and the Vironostika-Less Sensitive enzyme immunoassay (Vir-LS) were highly correlated in the context of other phases of infection, not just in elite controllers [26]. Additionally, results from 180 HAART-suppressed patients reveal a steady decrease in Vir-LS values for the twelve months after viremia became undetectable [27]. In contrast, the avidity assay used in our study did not misclassify elite suppressors as recently infected; this was confirmed using two assay platforms as the basis for the avidity assay (BioRad HIV-1/HIV-2 EIA and BioRad HIV-1/HIV-2+ O EIA). In a previous study, patients who initiated ART during the acute phase of HIV infection had muted serologic responses to HIV infection [28], which could potentially impact results obtained with an antibody avidity; we did not evaluate patients with early ART initiation in this study.

The changes we observed in BED-CEIA values with viral suppression are consistent with results from previous studies [29]. For example, in the Multicenter AIDS Cohort Study (MACS), men who initiated ART had an average decrease of 0.54 OD-n and that the longer patients are receiving ART the more likely they were to be misclassified as recent infection using the BED-CEIA. A recent study in South Africa found that the proportion of patients misclassified by the BED-CEIA increased with the duration of ART [30]. Our study observes that the ART-induced viral suppression results in decreased BED-CEIA OD-n values in most patients. Additionally in most patients, when the virus rebounds, our data suggests that the immune system responds by increasing the proportion of antibody that is specific for HIV. For other assays that identify recent HIV infection based on the quantity of anti-HIV antibodies (e.g. titer-based assays), the presence or absence of circulating virus may also impact the rate of false-recent misclassification.
